# Rectification of Erroneous Chronological Dating of Three Anecdotal Events Occurring in the Early History of Radiology in Berlin

**DOI:** 10.5334/jbsr.3719

**Published:** 2024-09-30

**Authors:** Jean-François Monville, Robert-F. Dondelinger

**Affiliations:** 1 Department of Medical Imaging, St. Nikolaus Hospital, Eupen, Belgium; 2 Department of Medical Imaging, St. Nikolaus Hospital, Eupen, Belgium

**Keywords:** W.C. Röntgen, Paul Spies, history, radiology, Berlin, 1896

## Abstract

The authors define corrected dates of three remarkable events of early history of radiology in Berlin, which have been wrongly reported in the literature. Compiled evidence from contemporary newspaper publications demonstrates that Röntgen delivered his lecture to kaiser William II on 12 January 1896. Paul Spies gave a lecture on Röntgen rays to the Reichstag on 13 February 1896. The first publicly taken X-ray in Berlin was obtained on 20 January 1896.

## Introduction

It is common knowledge that radiology came into existence with the discovery of X-rays by Wilhelm Conrad Röntgen (1845–1923) on 8 November 1895 in Würzburg, Germany [1]. Accurate dating of historical episodes, including those which occurred in the early days of radiology, should be indisputable, considering the huge amount of evidence assembled by scientific articles, editorials, books, conference reports, newspaper accounts, and contemporary witnesses. A checklist reckons about 1000 contributions related to Röntgen rays, published during the sole year of 1896 [2]. However, doubtful provenance of information, nonexhaustive search for primary sources or simply transcription errors might result in improper dating, henceforth reproduced by ensuing writings. Moreover, inaccuracies committed by recognized authorities are perpetuated with only an outside chance of ever being questioned.

We report three examples of erroneous dating of anecdotal historical events which occurred in Berlin during the first 6 weeks following the announcement of the discovery of the Röntgen rays. Experimentation with the new rays was particularly acute in Berlin, starting as soon as the news of the discovery was spread during the first half of January 1896.

## Wilhelm Conrad Röntgen Lectured at the Imperial Court of Berlin on 12 January 1896

**Figure 1:**
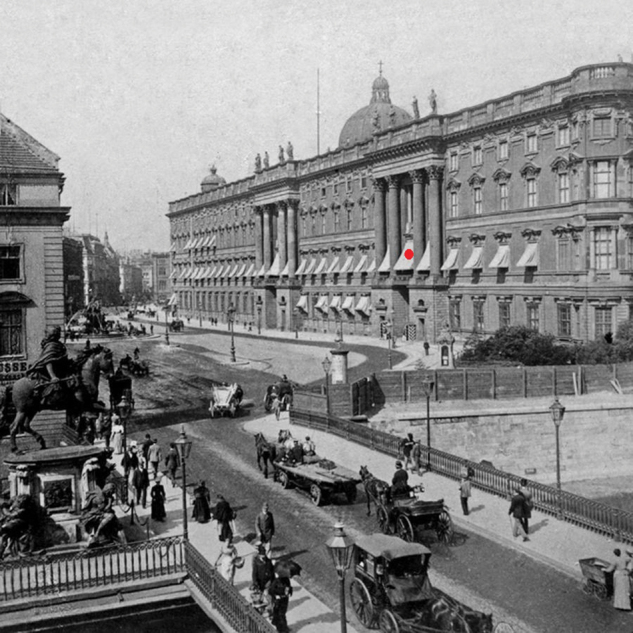
South front of the city palace in Berlin around 1900. The chamber of stars was located on the first floor above gateway number 1 (red dot).

On 1 January 1896, Röntgen sent an offprint of his article entitled “On a new kind of rays. Preliminary communication” and nine attached photographs to Emil Warburg (1846–1931), professor of physics at the University of Berlin. Warburg acknowledged receipt of the mail on 3 January and announced his intention to exhibit Röntgen’s material the next day at the university Institute of Physics, on the occasion of the 50th anniversary celebration of the physical society of Berlin [3]. Röntgen’s photographs went largely unnoticed by the unprepared minds of the organizers and delegates present at the solemn meeting. Anna von Mohl (1834–1899), the widow of the physicist Hermann von Helmholtz (1834–1894) and a glittering *salonnière*, attended the jubilee as a distinguished invited guest. Having picked up on Röntgen’s breakthrough, she alerted her acquaintance, empress dowager Frederick (1840–1901), who showed a keen interest in scientific novelties. The empress instructed Wilhelm von Bezold (1837–1907) director of the royal Prussian meteorological institute at the university and president of the physical society of Berlin (1895–1897) to collect more information on the nature of the new rays. In a letter to Röntgen dated 7 January 1896, von Bezold requested a photograph of the* “*human hand skeleton, the galvanometer in a box, and the Stanniol stripes” for presentation to the empress [4]. The galvanometer was, in fact, a compass. On 7 and 8 January, the Frankfurter Zeitung und Handelsblatt [5, 6] echoed the news of the discovery of X-rays, heralded by the Viennese journal Die Presse on 5 and 7 January [7, 8]. Kaiser William II (1859–1941) was certainly briefed by his mother and learned about the invisible rays from the local press as early as 8 January [9–13]. It can be assumed that the personal interest of the kaiser in the Röntgen rays was triggered by the deformity of his left arm, caused by brachial plexus elongation during delivery. Indeed, a radiograph of the imperial segmental skeletal anatomy was taken later during 1896. William II summoned Röntgen to Berlin by cable to perform a replication of his experiments at Court. In a telegraphic reply from 10 January, Röntgen wanted to know “Your Majesty may highly inclined command when I should come.” [14] Röntgen rushed to the Reich capital by train, all matters ceasing, in company of his assistant Otto Friedrich Stern (1868–1902), apparently without awaiting further instruction from the palace. He took up his quarters at the recently opened upper class Hotel Reichshof, Wilhelmstrasse, 70a, where he was reached by a telegram issued on Saturday, 11 January at 17:15 by the aide-de-camp on duty Hans Ferdinand Gustav von Arnim (1846–1922). The message was initially cabled to professor “Konrad Roentzgen” (sic) in Würzburg, and from there, it was forwarded to hotel Reichshof. The timing suggests that Röntgen had more likely left Würzburg on Saturday, 11 January rather, than 10 January, for a journey that lasted a great part of the day. The instruction read “His majesty deigns to receive your high born’s lecture tomorrow on Sunday at 5 o’clock at the chamber of stars of the local palace.” [15] (Figure 1) (Sunday meant 12 January). The newspaper Der Reichsbote inaccurately reported that Röntgen’s demonstration materialized at the new palace in Potsdam [16]. Otto Glasser (1895–1964), the world’s authority in Röntgen’s biography, assigned the date of Monday, 13 January 1896 to the imperial lecture, without quoting a source [2 bis]. This erroneous date has been accepted and unquestioned by all German or English reeditions of Röntgen’s biography, despite a corrective article being published in 1964, which continues to be ignored [17]. On the following days, local newspapers reported the experimental demonstration at the city palace by similar wording, Röntgen “demonstrated experiments and showed photographs” [18–24]. The entourage of the kaiser attending the X-ray session was explicitly named: empress Augusta Victoria (1881–1921), empress Dowager Frederick, Dr. Julius Robert Bosse (1832–1901), Prussian Minister of Educational and Religious Affairs, Dr. Hermann von Lucanus (1931–1908), chief of the secret civil cabinet of the emperor, and Professor Dr. Rudolf Leuthold (1832–1905), personal physician of William II. Members of the royal suite were in waiting. At the end of the dinner following the lecture, the emperor bestowed the Order of the Crown second class upon Röntgen. In its issue of Tuesday, 14 January, the Volks-Zeitung gave a detailed report of the lesson. Röntgen’s assistant was mistakenly named “Dr Stein,” and the date of the lecture was incorrectly labeled “yesterday afternoon,” i.e., 13 January [25]. The Berliner Abendpost of 14 January 1896 also reported erroneously that Röntgen gave his lecture “yesterday afternoon” [26]. These errors resulted most likely from heedless copyediting. On a later page of his book, Glasser refers once more to the date of 13 January, in relation with an article published by the Kieler Neueste Nachrichten or General-Anzeiger für Schleswig-Holstein, dated 4 February 1896 [2 ter]. This column reproduced in extenso the content of a private letter of unrevealed origin, owned by the Kölnische Zeitung. It translates in a bloomy style the scenario of Röntgen’s discourse held at the palace. The letter stated that Röntgen gave his lecture on the day of his arrival in Berlin, which is contradicted by the issue-date of the telegram cabled by the palace on Saturday 11 January. The paper also said that members of His Majesty’s General Headquarters of Berlin attended, which led to the assumption that Helmuth Johannes Ludwig von Moltke (1848–1916), at that time a colonel, aide-de-camp to the emperor and future chief of the General Staff during World War One, was present at Röntgen’s lecture. Moltke was not expressly mentioned by the press, as even if he was present, he would have been part of the suite of the emperor and not an outside guest.

All in all, the telegram issued by the entourage of the emperor and the newspaper reports offer ineludible proof that Röntgen delivered his lecture at the city palace of Berlin on Sunday, 12 January. Röntgen himself confirmed in a letter to the emperor dated 12 January 1897 that he gave his lecture at the palace “today, one year ago” [27]. The couple of newspapers suggesting the wrong date of 13 January 1896 might be the origin of the confusion appearing later in the literature and the error ingrained in Röntgen’s biography by Glasser.

## Paul Spies took the First Public X-Ray in Berlin on 20 January 1896

Amateur and artistic photography was a favorite pastime in Berlin at the turn of the nineteenth century. About seventy photographic workshops were registered Unter den Linden*.* Several photographic associations functioned in the Reich capital. Illustrated periodicals, exclusively dedicated to photography were published. The recently discovered invisible X-ray light sparked overwhelming interest among the community of photographers. For the purpose of the study of the X-rays, an association for scientific photography was established in Berlin. The members met for the second time on 30 January at 19:00 at the photochemical laboratory of the royal technical high school in Berlin-Charlottenburg [28]. Another club, the German association of friends of photography, founded in 1887 [29], called for a meeting the day before, at the academy of war, Berlin-Mitte, Unter den Linden 70–74/Dorotheenstrasse 58–59.

A recently published article ascertained without proof that the first publicly demonstrated X-ray in Berlin was obtained during this particular meeting on 29 January 1896 [30]. For the occasion, the physicist professor Eugen Goldstein (1850–1930), assistant at the new royal observatory of Berlin-Kreuzberg and one of the managers of the association for scientific photography, gave an experimental lecture on Röntgen rays. He took an X-ray of the hand of Marie Kundt (1870–1932), the niece of the physicist August Kundt (1839–1894), professor of physics in Berlin, predecessor of Emil Warburg and mentor of W.C. Röntgen in Zürich and Strassburg. In 1896, Marie Kundt was an assistant at the Lette photographic teaching institute in Berlin, Viktoria-Luise Platz 6, founded in 1866*.* She later became the director of the house and played an important role in the training of female technical assistants in photography and radiography. The director in office, Dagmar Carl Siegbert Schultz-Hencke (1857–1913) attended Goldstein’s lecture as a cospeaker. He explained the X-ray photographs obtained by Röntgen in Würzburg, which were shown to the floor. He also supervised the exposure and processing of the X-ray taken during the session. The full house was attended by officers of all branches of service among other guests [31, 32]. Although the anecdote is a picturesque one, it is irrelevant for the history of radiology. Nevertheless the same diligence should be practiced in dating priorities of small happenings as of big ones. The date of 29 January 1896 attributed to the first X-ray publicly taken in Berlin is incorrect.

**Figure 2:**
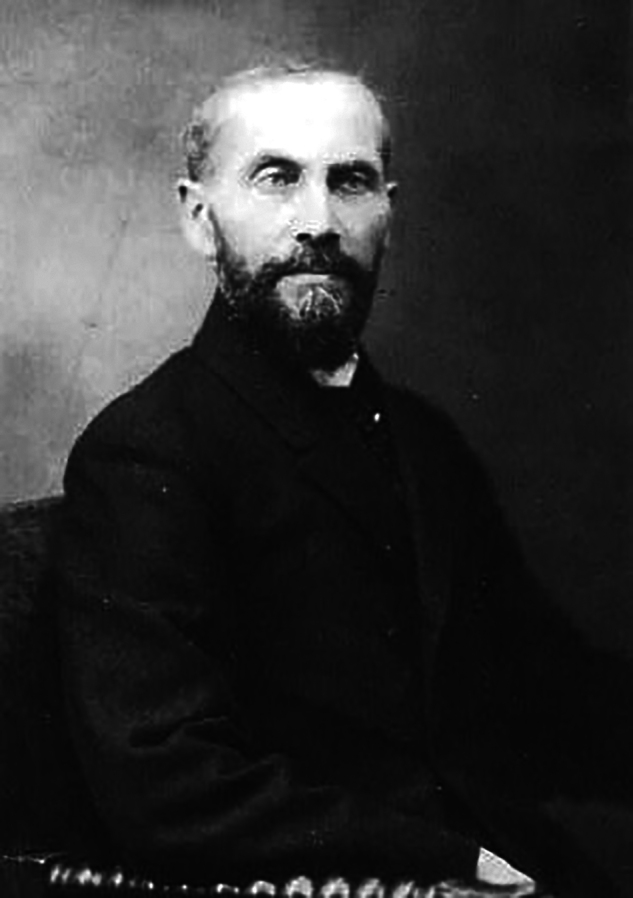

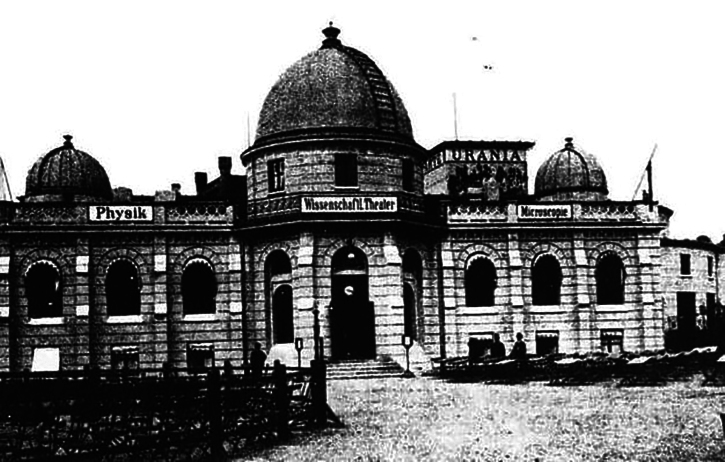
(above) Paul Spies (1862–1925). In 1896, he was a physicist at the Urania, Berlin. (below) Urania institution, Invalidenstrasse 58, Berlin in 1900 [33].

In 1896, Paul Spies (1862–1925), a physicist and early pioneer of fluoroscopy and roentgenography, was the head of the section of physics at the Berlin Urania institute, Invalidenstrasse, 58. The Urania association was founded on 3 March 1888; its home building was inaugurated on 1 July 1889 [33] (Figure 2). The aim of the thriving institution was to communicate the most recent scientific discoveries to a broad public. Regular lectures were given in a theater with a balcony, comprising 500 seats. As soon as Spies gained satisfactory experience with Röntgen rays around the second half of January 1896, he delivered experimental lectures on the new rays at the Urania. The journal Der Reichsbote of Thursday, 16 January [34] and the official imperial gazette Deutscher Reichs-Anzeiger und Königlich Preussischer Staats-Anzeiger of Saturday, 18 January [35] announced the first public experimental lecture on X-rays, entitled “Photography with invisible rays,” scheduled on Monday evening, 20 January. The conference followed in a row a lecture on another topic starting at 19:00. A detailed commentary of this noteworthy event was published by the Deutscher Reichs-Anzeiger in the issue of Tuesday, 21 January [36]. The article reported that Spies took a radiograph of a leather wallet containing a small key and two coins. The wallet was wrapped with black fabric and covered by a fingerbreadth wooden board during time of exposure to the X-rays. Another journal echoed the event in similar terms [37]. Unfortunately, the original X-ray photograph has until now remained lost; although, similar roentgenographs obtained during the same time period have been preserved. Paul Spies continued delivering experimental lectures on Röntgen rays at the Urania during the first months of the year 1896. The weekly lectures were advertised in the previous Saturday issues of the Deutscher Reichsanzeiger and by other local newspapers.

The advertisement by the local newspapers of the public conferences scheduled at the Urania Institute and the account given by the press establish inevitably that the first public X-ray demonstration in Berlin took place at the Urania on Monday evening, 20 January 1896.

## Paul Spies Demonstrated the X-Rays at the Reichstag on February 13, 1896

**Figure 3:**
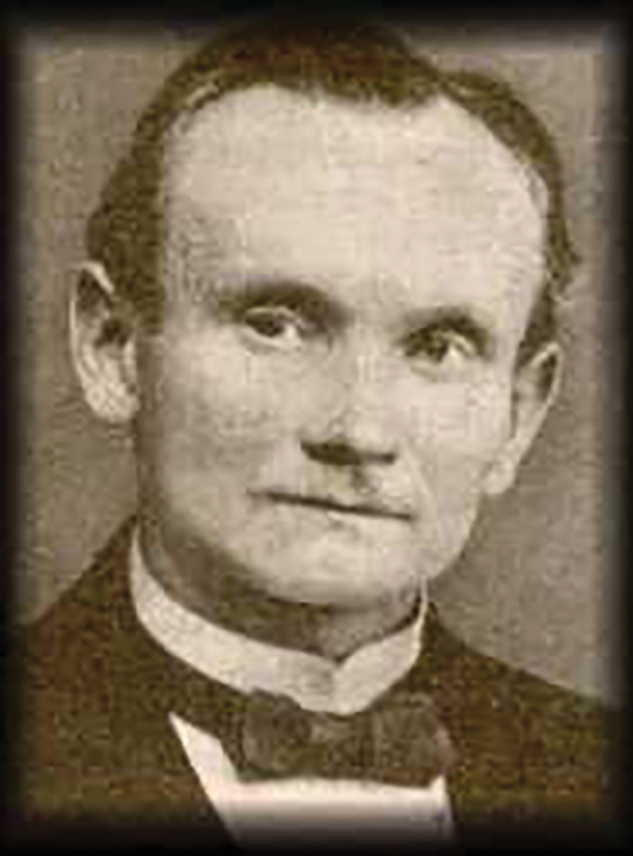
Reinhold Burger (1866–1954). In early 1896, he was a pioneer in manufacturing X-ray tubes in Berlin [47].

**Figure 4:**
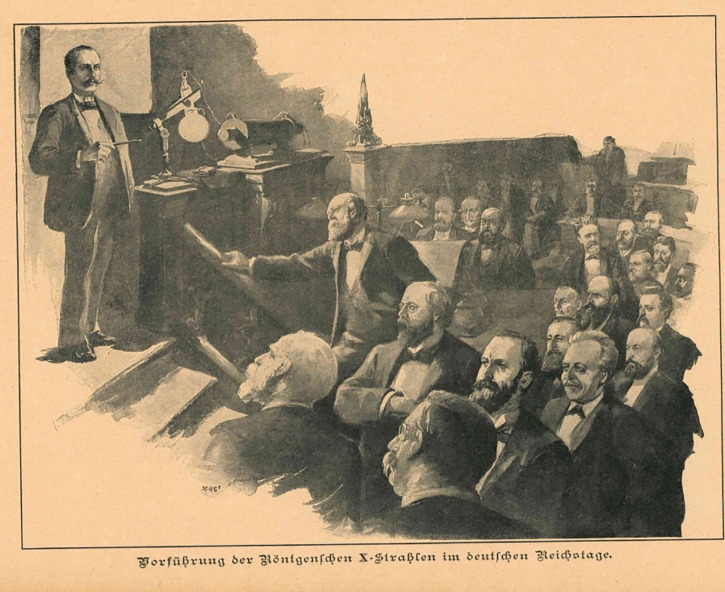
Drawing illustrating the lecture given by Paul Spies at the Reichstag on 13 February 1896 [49].

**Figure 5:**
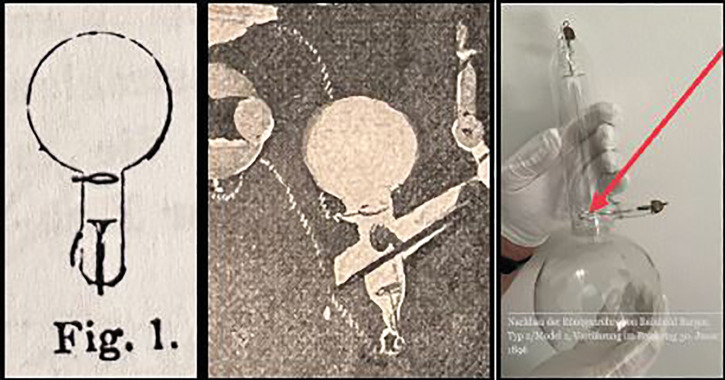
(left) Drawing by Paul Spies of the X-ray tube with a circular anode designed by Reinhold Burger [48]. (middle) Magnified view of the circular anode of the X-ray tube shown in Figure 4 [49]. (right) Rebuilt original X-ray tube of Reinhold Burger, in regular use by Paul Spies [47].

**Figure 6:**
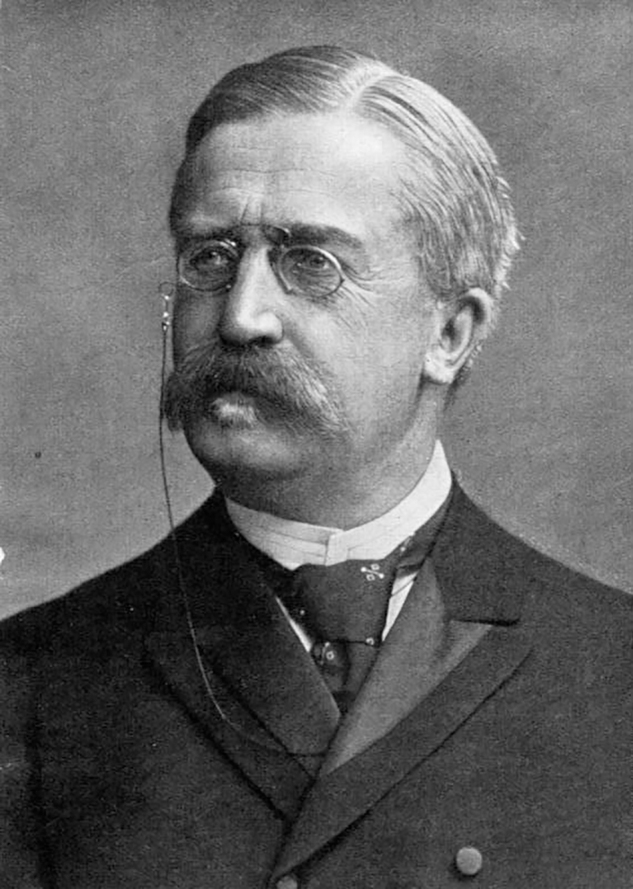
Karl Heinrich von Bötticher (1833–1907). Photograph between 1880 and 1900. https://www.bild.bundesarchiv.de/dba/de/ Bild 146-2008-0022.

At an unknown date following the lecture presented by Röntgen at Court, baron Rudolf von Buol-Berenberg (1842–1931), president of the Reichstag from 1895 to 1898, invited the Würzburg professor to repeat his experimental demonstration to the high assembly. As Röntgen declined, von Buol-Berenberg had to search for a replacement. Professor Goldstein was not a conceivable choice due to strong antisemite hostility spreading in some political circles of the Reichstag. The president approached the Urania association and Spies was appointed as a surrogate for Röntgen. Glasser affirms in Röntgen’s biography without quoting a source, that Spies gave his lecture to a full house on Thursday, 30 January 1896 [2 quater]. This date, which is invariably reproduced in all books, articles and internet sites dealing with the anecdote, is incorrect. A potential source for the origin of the wrong date is a notice published in an American technical journal that Otto Glasser might have consulted [38]; 30 January does not comply with the agenda of Spies, who was out of town at the end of the month. Spies had responded positively to an invitation issued by the Viennese association of technicians in electronics (Elektrotechniker Verein) presided by Ludwig Boltzmann (1844–1906), professor of theoretical physics at the university of Vienna. On 30 January Spies gave two lectures in Vienna, one entitled “Modern Illumination,” which delt with Tesla’s cold light and another “On Röntgen rays.” Spies has recently given these same lectures at the Urania. The next day, on 31 January, Spies talked on “The manifestation of high voltages.” The journal of the association of Austrian electrotechnicians gave a detailed account of the conferences presented by the invited guest speaker from Berlin [39]. For the practical experiment, Spies took along the cathode tube which he used during the Urania lectures. The tube was heated up for 14 min before taking an X-ray of a wallet which contained a key and was covered with a wooden board. The photographic plate was wrapped with black paper. Spies also demonstrated the fluorescence of a layer of barium platinocyanide painted on a piece of cardboard and excited by the X-rays. The speaker evidenced the skeletal shadows of an interposed hand projected on the illuminated surface. Back home in Berlin, Spies resumed his lectures on Röntgen rays at the Urania on 3–5, 7, 10, 12, 14–15, 17–19, and 25–27 February and went on lecturing during the next month [40–43]. It can be estimated that in Berlin around 10,000–14,000 spectators witnessed the public demonstration of X-rays by Spies during the first trimester of 1896. Among the few dates still left open in Spies’ agenda, Thursday 13 February 1896 was chosen for the memorable speech. The corresponding journalist of The Journal (New York) cabled from Berlin on 16 February that “von Buol-Berenberg had issued invitations to the members of the Reichstag, the Bundesrath and the press last week.” [44] The Reichstag was fully packed on Thursday night. Ladies occupied the balcony. Many high ranking politicians attended: Karl Wilhelm von Bötticher (1833–1907), State Secretary for Internal Affairs, Karl Heinrich von Schönstedt (1833–1924), Prussian Minister of Justice, Adolf, Baron Marschall von Bieberstein (1842–1912), State Secretary for Foreign Affairs, and Friedrich von Hollmann (1842–1913), State Secretary of the Imperial Office of the Navy. The president von Buol-Berenberg was half an hour late [45, 46]. During the day, the members of the Reichstag discussed the financial forecast of the Ministry of Foreign Affairs in the same great hall until 17:00. Spies’ lecture lasted from 21:00 to 22:30. For the experimental part, Spies used a particular cathode tube named model-2 or type-2 X-ray tube, conceived by the Berlin X-ray pioneer Reinhold Burger (1866–1954) and manufactured at his workshop Novalisstrasse, 6 [47] (Figure 3). Burger’s X-ray tube was unmistakably characterized by a circular shaped anode, which Spies represented in a small drawing in the published material covering his lectures on Röntgen rays at the Urania [48]. Recently, Mr. Sven Burger, the grandson of the inventor, had the original tube rebuilt by a German glass blowing company. A singular drawing exists of the X-rays at the Reichstag and is kept in the archives of the German Röntgen museum (Figure 4). It features Spies standing on the speaker’s platform delivering his lecture. The so far occult origin of this drawing was recently uncovered by Mr. Marcel Michels, archivist of the German Röntgen museum [49]. Spies successfully took an X-ray of a leather wallet, which contained a small key offered by Max Liebermann von Sonnenberg (1848–1911), leader of the antisemite German social reform party (Deutschsoziale Reformpartei, DSRP). Spies added a 10-Pfennig coin. During the interlude, while waiting for the X-ray to be processed, the members of the Reichstag flocked close to the podium and showed great interest in the cylinder shaped fluoroscopy device conceived by Spies, similar to the kryptoscope designed by the physicist Enrico Salvioni in Italy. The drawing is of exquisite finesse in depicting details. It reproduces the small annular anode of the Burger-X-ray tube and shows the wallet placed under the tube (Figure 5). Among the prominent public civil servants attending, Karl Wilhelm von Bötticher is recognized in the foreground (Figure 6).

There is inescapable evidence from Spies’ agenda and published Austrian and German material that the X-ray demonstration at the Reichstag by Spies took place on Thursday, 13 February 1896.

## Conclusions

Pioneering research in X-rays became particularily intense in Berlin after the announcement of the discovery of Röntgen’s invisible light. The physicists Eugen Goldstein and Paul Spies, among others, played a determinant role in investigating the physical and photographic properties of the Röntgen rays in the early days in Berlin. The large number of local daily newspapers makes it easy to put a date on anecdotal historical events that took place in the Reich capital at the turn of the nineteenth century. In the authoritative biography of Röntgen by Otto Glasser, the dates of the demonstration of the rays by Spies to the kaiser and the Reichstag are incorrect. Our research has allowed us to allocate the correct dates respectively of 12 January and 13 February 1896 to these events. A recently published paper affirmed that Goldstein obtained the first X-ray in public in Berlin on 29 January 1896. In fact, it was Spies at the Urania on 20 January 1896.
